# Antioxidant, antidiabetic and hypolipidemic effects of *Tulbaghia violacea* Harv. (wild garlic) rhizome methanolic extract in a diabetic rat model

**DOI:** 10.1186/s12906-015-0932-9

**Published:** 2015-11-17

**Authors:** Kogi Moodley, Kimane Joseph, Yougasphree Naidoo, Shahidul Islam, Irene Mackraj

**Affiliations:** Department of Human Physiology, School of Laboratory Medicine and Medical Sciences, College of Health Sciences, University of Kwazulu Natal, Durban, 4000 South Africa; Department of Biological & Conservation Sciences, School of Life Sciences, University of Kwazulu Natal, Durban, 4000 South Africa; Department of Biochemistry, Genetics and Biochemistry, School of Life Sciences, University of KwaZulu-Natal, Durban, 4000 South Africa

**Keywords:** *Tulbaghia violacea*, Streptozotocin, Diabetes, Hypoglycaemia, Oxidative stress, Lipid

## Abstract

**Background:**

The prevalence of diabetes mellitus (DM) continues to rise alarmingly despite years of intensive research. The need to explore alternative remedies such as traditional phytotherapy has therefore become increasingly important in the management and treatment of DM.

**Methods:**

Diabetes was induced by a single intraperitoneal (i.p) injection of streptozotocin (40 mg/kg.b.w) in male Wistar rats. The rats were divided into 5 groups as follows: non-diabetic control fed distilled water, diabetic control fed distilled water, diabetic group treated with *Tulbaghia violacea* (TVL) (60 mg/kg.b.w), diabetic group treated with TVL (120 mg/kg.b.w), and diabetic group treated with glibenclamide (10 mg/kg.b.w). Food and water intake, as well as urine output were measured daily, whilst body weight and fasting blood glucose were monitored weekly. On day 42, an oral glucose tolerance test was performed on all groups. After 7 weeks, the animals were sacrificed by halothane overdose, blood was removed by cardiac puncture and tissues were harvested. Assays were performed for the determination of plasma insulin, liver glycogen content, lipid peroxidation, antioxidant enzyme levels, plasma nitric oxide levels and serum lipid and liver enzyme levels.

**Results and Discussion:**

TVL treatment improved body weights, significantly reduced fasting blood glucose levels, improved glucose tolerance and significantly increased plasma insulin and liver glycogen content. TVL treatment also reduced liver thiobarbituric acid reactive substances (TBARS) levels, increased liver superoxide dismutase (SOD), catalase and glutathione peroxidase (GPx) and increased plasma nitric oxide (NO) levels. Furthermore, TVL administration reduced serum triglycerides, VLDL, total-cholesterol levels and increased HDL-cholesterol levels. TVL also reduced serum levels of liver enzymes, alanine aminotransferase (ALT) and aspartate aminotransferase (AST).

**Conclusion:**

Data obtained in this study demonstrated the hypoglycemic, antioxidant, hepatoprotective and hypolipidemic effects of TVL in STZ-induced diabetic rats.

## Background

Diabetes mellitus (DM), one of the most challenging health pandemics of the 21st century currently affects about 347 million people worldwide [1]. This number is rapidly increasing and is expected to double by the year 2030, making diabetes the 7th leading cause of death in the world. DM is a complex metabolic disorder, characterized by high blood glucose levels (hyperglycaemia) and impaired lipid, carbohydrate and protein metabolism as a result of defects in insulin secretion, insulin action or both [2, 3]. As a consequence of these metabolic alterations, at least 50 % of people with diabetes develop one or more microvascular (viz. retinopathy, neuropathy and nephropathy) or macrovascular (viz. myocardial infarction, heart failure and stroke) complications [2]. These complications are common to both types of diabetes, despite the difference in the pathogeneses of the diseases [3].

Poor control of blood glucose levels is the key contributing factor to the associated complications and treatment of hyperglycaemia is therefore, the main target in the prevention of these diabetes- related complications [4, 5]. Hyperglycaemia plays a critical role in the development and progression of diabetic complications by numerous mechanisms, including increased oxidative stress, decreased nitric oxide bioavailability, glucose autoxidation and non-enzymatic protein glycation [6]. It is also well known that oxidative stress develops when reactive oxygen-derived free radical production exceeds the antioxidant defense mechanism of the cell [6, 7]. DM has been shown to be associated with increased free radical formation and decreased antioxidant capacity, leading to oxidative damage to lipid, carbohydrate, protein and nucleic acids [6]. Antioxidants decrease diabetic complications by attenuation of free radical associated damage [7]. Furthermore, altered levels of blood lipids (dyslipidemia), mainly triglyceride and cholesterol are involved in the pathogenesis of cardiovascular disease in both type 1 and type 2 diabetes [2, 3]. The detection and treatment of dyslipidemia could, therefore reduce the risk of cardiovascular disease and its consequences in diabetic patients [2].

Current therapies used in the treatment of DM include, insulin and pharmaceutical oral hypoglycaemic/antidiabetic agents such as sulphonylureas, biguanides and glinides which have their own limitations and undesirable side-effects such as hypoglycaemia, gastrointestinal disturbances, lactic acidosis and liver toxicity [8–10]. In recent years, the search for alternative therapeutic agents in the treatment of diabetes has been the focus of scientific research. Notably in 1980 the WHO Expert Committee on DM recommended further scientific investigations into the therapeutic efficacy of medicinal plants due to their perceived minimal side-effects. Many herbal medicines and medicinal plants with diverse actions have been used traditionally for the control, management and/or treatment of DM in many parts of the world [3]. Consequently, screening of medicinal plants for therapeutic purposes, is important in drug development as they may possess hypoglycaemic, hypolipidemic and antioxidant activities which may be effective in the treatment of diabetes mellitus [10, 11].

*Tulbaghia violacea* is an herbaceous plant, indigenous to southern African countries particularly in the rocky grasslands of the Eastern Cape, Southern KwaZulu-Natal and Gauteng provinces of South Africa. It has been used in folkloric medicine since early times to treat a number of human diseases and ailments, including hypertension, asthma, gastro-intestinal disorders, esophageal cancer, fevers and colds, inflammation, tuberculosis and bacterial infections [12–15]. It has a garlic-like odor (alliaceous odor) that is released when the leaves or the bulbs are bruised or damaged [16] and is commonly known as “society garlic”, “sweet garlic” and “wild garlic” [12, 15, 17, 18]. *T. violacea* has been shown to have antihypertensive as well as antioxidant effects in various rat models [19–24]. Previous studies have demonstrated the blood pressure lowering effects of *T. violacea* in Dahl salt-sensitive (DSS) rats [19, 21]. It also showed increased antioxidant activity and decreased lipid peroxidation in rats fed *T. violacea* methanolic extracts [25]. It is well known that oxidative stress is implicated in the development of various pathological conditions including hypertension and diabetes. Hence, the present study was designed to examine the effects of *T. violacea* methanolic extract on blood glucose, serum lipids and anti-oxidative status in streptozotocin-induced diabetic rats.

## Methods

### Chemicals

Streptozotocin was obtained from Sigma Aldrich (St. Louis, MO, USA). Sandoz Glibenclamide was obtained from Sandoz SA (Pty) Ltd. (Gauteng, South Africa). All other chemicals used were purchased from Merck Chemicals (Germany).

### Plant material

*Tulbaghia violacea* rhizomes were collected in Durban, Kwazulu Natal, identified by botanist, H. Baijnath and a voucher specimen was deposited in the Ward herbarium at the University of Kwazulu Natal, Durban, South Africa.

### Preparation of extract

The rhizomes were washed thoroughly under tap water and air dried at room temperature for 48 h. Thereafter, the plant material was weighed, crushed in a Waring blender, immersed in methanol and agitated on a shaker for 48 h. The crude extract was then filtered using Whatman filter paper and the filtrate was concentrated in a rotary evaporator (Heidolph, Schwabach, Germany). The extract was freeze-dried and stored in a desiccator at −4 °C for later use. The methanolic extract yield was calculated as 1.01(g/g).

### Phytochemical analysis of *Tulbaghia violacea*-Gas chromatography–mass spectrometry (GC-MS)

Analysis by GC–MS was carried out using Perkin-Elmer^®^ Gas Chromatography (Clarus^®^ 580) equipped with MSD mass spectrometer (Clarus^®^ SQ8S) instrument with built–in autosampler. The sample was analysed on Elite-5MS (30 m x 0.25 mm id x 0.25 μm) column. The oven temperature was programmed from 37 to 320 °C at a rate of 18-25 °C/min and held for 0.5 and 1.85 min at 18 and 320 °C, respectively. The injector temperature was 250 °C and MS Ion Source temperature was 280 °C with full scan and solvent delay of 0–2.30 min. MS Scan Range was m/z 35–500 in 0.10 s. One microlitre of the samples was injected in Helium carrier gas at split flow of 20 ml/min.

### Animals

Male Wistar rats (250–300 g) were obtained from the Biomedical Resource Unit (BRU) at the University of KwaZulu Natal, Westville campus, Durban, South Africa and kept under standard conditions (24 ± 1 °C, relative humidity 40–60 %, and 12/12 h light/dark cycle) for the 7 weeks experimental period. The animals had free access to food and drinking water during the entire experimental period. The protocol used in this study was approved by the University of KwaZulu-Natal Experimental Animal Ethics Sub-Committee (Ethical approval number 045/13/Animal).

### Induction of diabetes

Animals were fasted overnight and then injected intraperitoneally (i.p.) with a single dose of streptozotocin (40 mg/kg body weight) dissolved in freshly prepared 0.1 M citrate buffer (pH 4.5) [25]. Control rats were injected with citrate buffer alone. Seven days following the streptozotocin injection, blood was drawn from the tail vein and glucose concentration was measured using a portable glucometer (One Touch Select, Lifescan, Inc., CA. USA). Animals with a fasting blood glucose concentration >25 mmol/L were included in this study as diabetic rat.

### Study design

The rats were divided into 5 groups (*n =* 7) as follows:

- Group A : Non-diabetic control-received distilled water (3 ml/kg.b.w).
- Group B : Diabetic control- received distilled water (3 ml/kg.b.w).
- Group C : Diabetic - received TVL extract (60 mg/kg.b.w).
- Group D : Diabetic - received TVL extract (120 mg/kg.b.w).
- Group E : Diabetic - received Glibenclamide (10 mg/kg.b.w).

TVL and glibenclamide doses were administered daily via oral gavage. Food and water intake were monitored daily whilst body weights were determined weekly.

### Oral glucose tolerance test (OGTT)

An oral glucose tolerance test (OGTT) was performed on each rat 6 weeks after the intervention [26]. Following a single oral dose of glucose (2 g/kg.b.w), glucose concentrations were measured in the blood collected from the tail vein at 0 (just before glucose administration), 15, 30, 45, 60, 90, 120 and 180 min following the glucose ingestion.

### Animal sacrifice

Seven weeks after treatment, the animals were sacrificed by halothane anesthesia and blood was collected via cardiac puncture. The heart, kidney and liver tissues of each rat were harvested, weighed and snap frozen in liquid nitrogen. All biological samples were stored at −70 °C until further analysis. Sections of pancreas were removed and fixed in buffered neutral formalin for histological preparations.

### Determination of plasma insulin levels

Plasma insulin levels were measured by Enzyme-linked Immunosorbent Assay (ELISA) technique, using an Insulin (Rat) Ultrasensitive ELISA kit (DRG) Diagnostics (Marburg, Germany) [27].

### Determination of liver glycogen content

The liver glycogen content was measured according to a modified method described by Lo et al., 1970 [28].

### Histopathology of the pancreas

Portions of the pancreas were excised and fixed in buffered neutral formalin. The tissues were embedded in paraffin wax, sectioned at 5 μ m and stained with haematoxylin and eosin (H&E). Histological sections were examined and scanned using a Leica SCN400 scanner (Germany) for evaluation of morphology and morphometric analysis. Quantitative analysis of islets was performed according to a modified method of Masjedi et al., 2013 [29] as follows: The number of islets in 10 microscopic fields of 10 x 100 μ m was counted and the average number of islets for each group was calculated. Pancreatic islet size was evaluated by measuring the area (μ m^2^) occupied by each islet counted using the Leica SCN software and calculating the mean area per group.

### Determination of serum lipids, liver-function enzymes

Serum triglyceride, total cholesterol, HDL-cholesterol, aspartate transaminase (AST), alanine transaminase (ALT) and creatinine were determined using an Automated Chemistry Analyzer (LabmaxPlenno, Labtest, Lagoa-Santa, Brazil) [25]. Low density lipoprotein (LDL)-cholesterol concentrations were calculated according to the formula given by Friedwald et al., 1972 [30] as follows: LDL-cholesterol = Total cholesterol– [HDL-cholesterol + TG/5)] where TG/5 is equivalent to the amount of very low density lipoprotein (VLDL)-cholesterol.

### Determination of lipid peroxidation in liver

Lipid peroxidation was assessed by modified method of the thiobarbituric acid reactive substances (TBARS) assay [31]. Liver tissues (50 mg) were homogenized in 0.2 % H_3_PO_4_ (450 μ l) and centrifuged at 10000 rpm for 15 min at 4 °C. The supernatant was decanted into glass tubes, into which 500 μ l of 2 % H_3_PO_4_ was added, and vortexed. This was followed by the addition of 200 μ l 7 % H_3_PO_4,_ TBA/BHT (400 μ l) and1M HCl (200 μ l). The tubes were heated at 100 °C for 15 min and then allowed to cool to room temperature. Butanol (1500 μ l) was then added to each tube and vortexed. The top phase of each solution was transferred in triplicate to a 96-well plate and absorbance was measured at 532 nm and 600 nm on a Spectrostarnano plate reader (BMG LABTECH, Ortenberg, Germany). Concentration was determined according to the following formula: Concentration = [(Abs.532-Abs.600)/156]**x**1000.

### Determination of plasma antioxidant levels

The activities of superoxide dismutase (SOD), catalase and glutathione peroxidase (GPx) in liver samples were determined using Biovision (California, USA) assay kits according to the manufacturer’s instructions [32].

### Determination of plasma nitric oxide levels

Plasma nitric oxide (NO) levels were measured as an indirect indicator of reactive nitrogen species (RNS) in plasma. To perform this assay, 25 μ l of plasma was added in triplicate to the wells of a 96-well microtitre plate. Sodium nitrite (0 μM-200 μM) was used as standard in this assay and 50 μl of its each concentration was added in triplicate to the wells. This was followed by the rapid addition of 50 μl vanadium chloride (VCl_3_), 25 μl 2 % sulphanilamide (SULF) and 50 μl 0.1 % *N*-1-napthylethylenediamine dihydrochloride (NEDD) into each well. The plate was then incubated (37 °C) for 45 min under dark conditions and the optical density was read at 540/690 nm using a spectrophotometer (Spectrostarnano, BMG LABTECH, Ortenberg Germany). A standard curve was constructed using the results obtained from the sodium nitrite standard and the resultant NO concentration for each sample was determined by extrapolation.

### Statistical analysis

All data are expressed as mean ± SEM. Statistical differences between the groups were analyzed by Analysis of Variance (ANOVA) using Graphpad prism 5.0 statistical software, *p* values <0.05 were considered to be significant.

## Results

### GC/MS Analysis of methanolic extract of *Tulbaghia violacea*

**Fig. 1:**
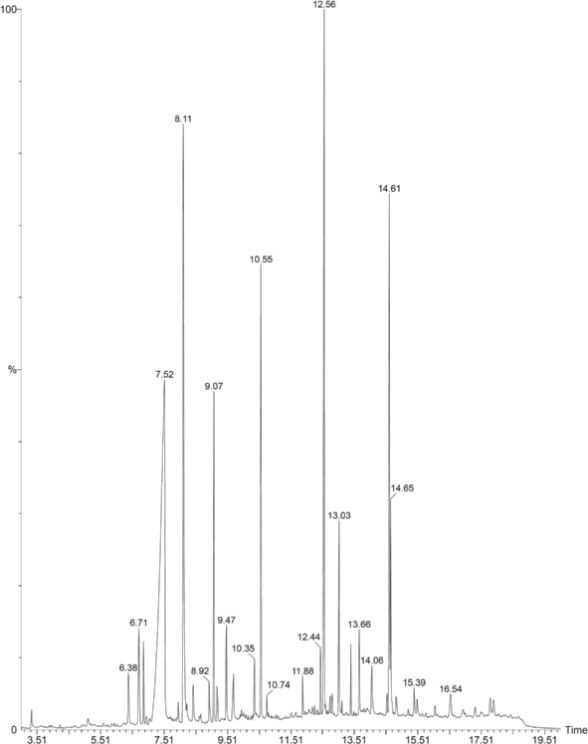
GC-MS chromatograph of methanolic extract of *Tulbaghia violacea* rhizomes

Qualitative analysis of the extract was performed using GC-MS (Fig. 1) and the individual constituents were identified by matching their mass spectra and retention indices with those of inbuilt citations library. The result of the analysis (Table 1) identified disulfides, 2, 4 - dithiapentane, n-propyl 9, 12-octadecadienoate, methyl 5, 13-Docosadienoate, amongst others.

**Table 1:** Compounds identified in GC-MS analysis of *Tulbaghia violacea* extract

S. No	Name of compound	RT	RI
1	Beta.-1,5-O-Dibenzoyl-ribofuranose	7.5	849
2	4-Methoxybenzaldehyde	8.1	927
3	Oleyl alcohol, trifluoroacetate	9.0	691
4	Disulfide, bis(2-sulfhydrylethyl)-	10.5	648
5	Benzene, 1-methyl-4-(methylthio)-	12.5	318
6	2, 4-Dithiapentane.	13.0	426
7	n-propyl 9,12-octadecadienoate	14.6	947
8	Methyl 5,13-Docosadienoate	14.66	690

### Food and water intake and body weight change

The average body weights, percentage left kidney weights (%LKW), and percentage heart weights (%HW) of the groups after 5 weeks of treatments are shown in Table 2. The pre-treated body weights of the diabetic, TVL 60 and TVL 120 experimental groups differed significantly from the non-diabetic control group, *p <* 0.05. There were no significant differences between the pre-treated body weights of the various diabetic experimental groups. There was a significant 19 % increase in the post-treated body weights (g) of NDC compared to the pre-treated weights, *p <* 0.05. D + TVL120 showed a significant 9 % increase in body weight when post-treated weight was compared to pre-treated body weight, *p <* 0.05. The post-treated body weights of the diabetic groups were significantly reduced compared to the non-diabetic control group, *p <* 0.001.

**Table 2:** Effect of TVL on pre-treated (baseline) and post-treated (week 7) bodyweights, relative organ weights and food and water intake in rats during the experimental period

Parameters	Experimental groups				
	NDC	DC	D + TVL60	D + TVL120	D + glibenclamide
Body weight (g)					
Pre-treated	334.40 ± 17.82	281.30 ± 9.07*	276.00 ± 10.54*	284.10 ± 6.38*	291.50 ± 15.20
Post-treated	411.00 ± 16.27^a^	287.60 ± 8.88***	295.20 ± 11.52***	312.90 ± 8.29^a^***	312.60 ± 12.71***
% HW	0.27 ± 0.022	0.35 ± 0.01***	0.32 ± 0.01^#^	0.30 ± 0.01^#^	0.32 ± 0.01^#^
% LKW.	0.25 ± 0.01	0.47 ± 0.01***	0.41 ± 0.01*** ^#^	0.38 ± 0.02*** ^#^	0.40 ± 0.01***^#^
% LLW	1.21 ± 0.09	1.75 ± 0.03***	1.65 ± 0.03***	1.57 ± 0.04** ^#^	1.55 ± 0.06** ^#^
Food intake (g/rat/day)	16.00 ± 1.47	37.00 ± 2.03**	34.75 ± 1.96**	30.00 ± 3.30^*^	36.67 ± 4.01**
Water intake (ml/rat/day)	18.00 ± 3.74	158.00 ± 5.83***	132.90 ± 7.78***^#^	111.70 ± 11.08***^#^	121.70 ± 7.03***^#^

Non-diabetic control (NDC) and diabetic control (DC); TVL 60 mg/kg.b.w.(D + TVL 60); TVL 120 mg/kg.b.w. (D + TVL 120) and glibenclamide 10 mg/kg.b.w. (D + glibenclamide. All data are expressed as mean ± SEM for 7 animals per group

^a^Significantly different from pre-treated weight, *p <* 0.05

*Significantly different from NDC, *p <* 0.05

**Significantly different from NDC, *p <* 0.01

***Significantly different from NDC, *p <* 0.001

^#^Significantly different from DC, *p <* 0.05

There was a significant increase in the %HW in the diabetic control rats compared to the non-diabetic control rats, *p <* 0.001. The %HW did not differ significantly when the TVL60, TVL120 and glibenclamide-treated rats were compared to the non-diabetic group. However, the TVL 60, TVL120 and glibenclamide treated animals showed significant reductions in %HW compared to the diabetic control group, *p <* 0.05. The %LKW increased significantly in all diabetic rats compared to the non-diabetic animals *p <* 0.001. The treated diabetic groups showed significantly reduced % LKW weights compared to the diabetic control group, *p <* 0.05. The percentage left liver weight (%LLW) was significantly increased in the diabetic control, TVL 60, (*p <* 0.001) as well as in the TVL 120 and glibenclamide-treated groups (*p <* 0.01), compared to the non-diabetic rats. The TVL 120 and glibenclamide-treated groups showed significant reductions in % LLW compared to the diabetic control group, *p <* 0.05.

The diabetic control, TVL60 and glibenclamide-treated rats showed significantly increased food consumption compared to the non-diabetic control group, *p <* 0.01. Food consumption in the TVL120- treated group did not differ significantly from the non-diabetic group. There were no significant differences between the diabetic control group and the diabetic treated groups. Water intake increased significantly in the diabetic groups, compared to the non-diabetic group, *p <* 0.001. The diabetic rats treated with both doses of TVL and glibenclamide showed significantly decreased water intake, compared to the diabetic control rats, *p <* 0.05.

### Effect of TVL on fasting blood glucose levels

**Fig. 2:**
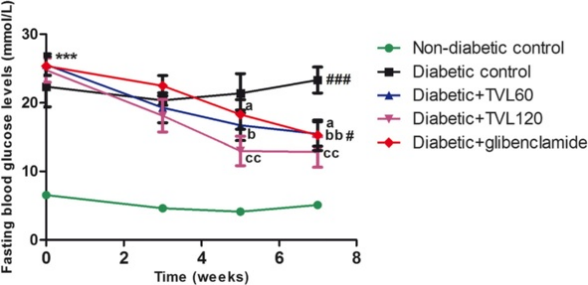
Effect of TVL on fasting blood glucose levels in non-diabetic and diabetic rats. All data are expressed as mean ± SEM for 7 animals per group. ***p < 0.001, significantly different compared to non-diabetic control. ^a^ *p <* 0.05 Significantly different from baseline value.^b^ *p <* 0.05 and ^bb^ *p <* 0.01, Significantly different from baseline value respectively. ^cc^ *p <* 0.01, Significantly different from baseline value. ^#^ *p <* 0.05 and ^##^ *p <* 0.01, significantly different from non-diabetic group at week 7

The effect of TVL on mean fasting blood glucose levels is shown in Fig. 2. Fasting blood glucose levels in the diabetic control, diabetic + TVL60, diabetic + TVL120 and diabetic + glibenclamide groups were significantly increased, compared to the non-diabetic group, *p <* 0.001. TVL (60 mg/kg.b.w) showed a significant reduction in fasting glucose levels at weeks 5 (35 %, *p <* 0.05) and 7 (40 %, *p <* 0.01), respectively compared to the baseline value. TVL (120 mg/kg.b.w) demonstrated a significant (48 %, *p <* 0.01) reduction in fasting glucose at weeks 5 and 7 compared to the baseline level. Glibenclamide treatment significantly reduced blood glucose levels at week 5 (28 %, *p <* 0.05) and week 7 (40 %, *p <* 0.05).

### Effect of TVL on OGTT

**Fig. 3:**
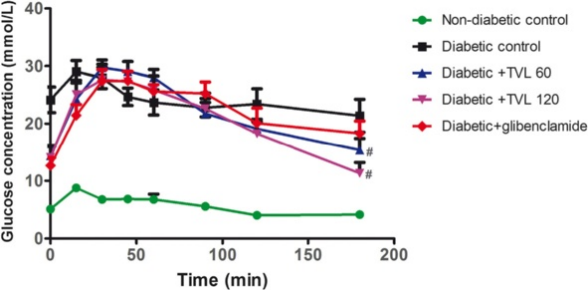
Effect of TVL on OGTT in non-diabetic and diabetic rats. All data are expressed as mean ± SEM for 7 animals per group. ^#^Significantly different from diabetic control, *p <* 0.05

**Fig. 4:**
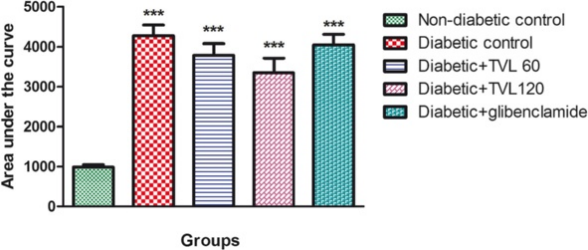
Area under the curve determined from OGTT graph of non-diabetic and diabetic rats. All data are expressed as mean ± SEM for 7 animals per group. ***Significantly different compared to non-diabetic control, *p <* 0.001

The effect of TVL on OGTT is shown in Fig. 3, whilst Fig. 4 represents the area under the curve (AUC). Glucose tolerance at 30 to 180 min was significantly improved in the TVL (60 and 120 mg/kg.bw) treated rat groups, compared to the diabetic control group (*p <* 0.05). The AUC_glucose_ value was found to be significantly increased in the untreated as well as in the treated diabetic groups compared to the non-diabetic control group (*p <* 0.001). There were no significant differences in the area under the curve between the diabetic groups, although there was a tendency towards a reduction in AUC_glucose_ in both TVL treated groups compared to the diabetic control group after 100 min.

### Effect of TVL on plasma insulin concentration

**Fig. 5:**
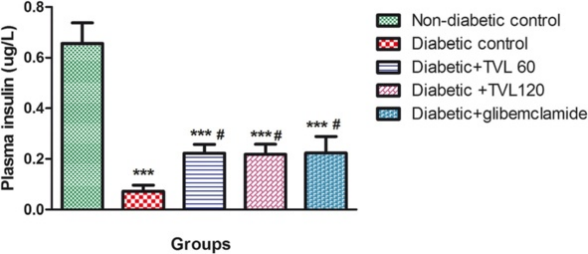
Effect of TVL on plasma insulin concentration in non-diabetic and diabetic rats. All data are expressed as mean ± SEM for 7 animals per group. ***Significant difference compared to non-diabetic control, *p <* 0.001. #Significant difference compared to diabetic control, *p <* 0.05

The plasma insulin levels in the various groups are shown in Fig. 5. Plasma insulin levels wereSignificantly reduced in the diabetic control and treated diabetic groups compared to the non-diabetic control group (*p <* 0.001). TVL and glibenclamide treatment significantly increased plasma insulin compared to the diabetic control group (*p <* 0.05).

### Effect of TVL on liver glycogen levels

**Fig. 6:**
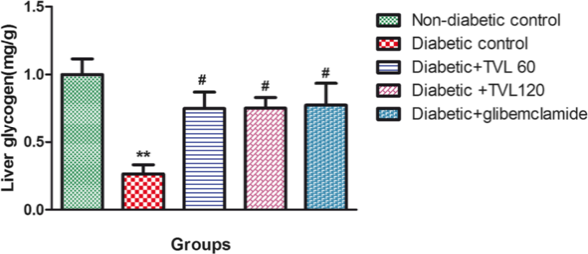
Effect of TVL on liver glycogen levels in non-diabetic and diabetic rats. All data are expressed as mean ± SEM for 7 animals per group. **Significant difference compared to non- diabetic control, *p <* 0.01. #Significant difference compared to diabetic control, *p <* 0.05

The liver glycogen levels in the various groups are shown in Fig. 6. The diabetic control group showed a significant 4-fold reduction in liver glycogen levels, compared to the non-diabetic control group (*p <* 0.01). The diabetic + TVL60, diabetic + TVL120 and diabetic + glibenclamide groups showed significant increases in liver glycogen levels compared to the diabetic control group (*p <* 0.05). There were no significant differences in the liver glycogen levels between the treated diabetic groups and the non-diabetic control group.

### Effect of TVL on histopathology of pancreatic Islets

**Fig. 7:**
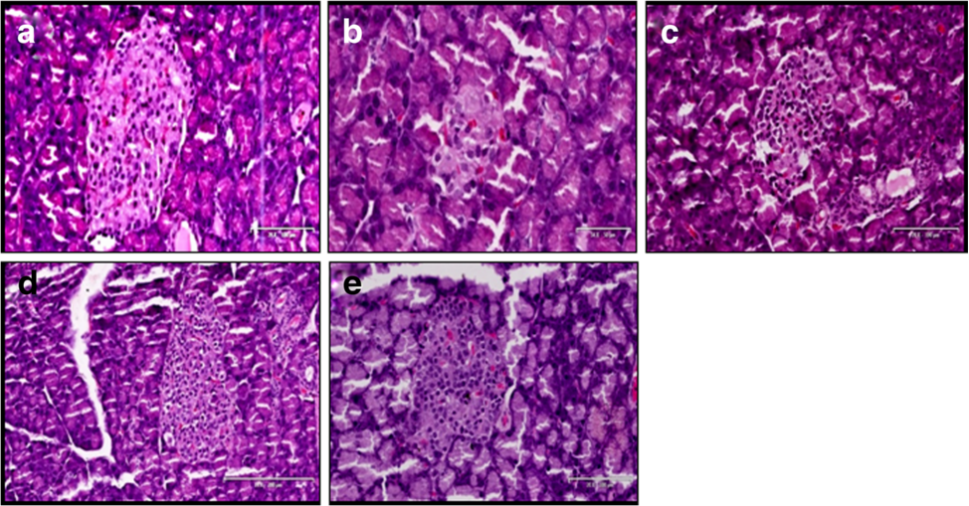
Photomicrographs of the pancreatic islets of normal and diabetic rats. **a** non-diabetic control; (**b**); diabetic control (**c**) TVL 60 mg/kg.b.w. **d** TVL 120 mg/kg.b.w; and (**e**) glibenclamide 10 mg/kg.b.w. Images taken at 20–100 μ m

**Fig. 8:**
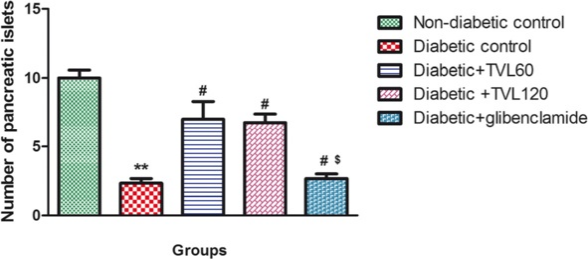
Effect of TVL on pancreatic islet numbers in non-diabetic and diabetic rats. All data are expressed as mean ± SEM for 7 animals per group. **Significantly different from non- diabetic control, *p <* 0.01. ^#^Significantly different from diabetic control, *p <* 0.05. ^$^Significantly different from TVL120, *p <* 0.05

**Fig. 9:**
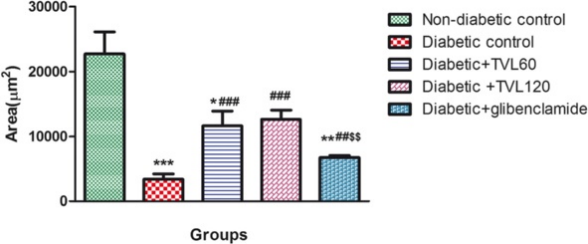
Effect of TVL on Area (μ m2) of pancreatic islet in non-diabetic and diabetic rats. All data are expressed as mean ± SEM for 7 animals per group. *Significantly different from non- diabetic control, *p <* 0.05. **Significantly different from non-diabetic control, *p <* 0.01. ***Significantly different from non-diabetic control, *p <* 0.001. ^###^Significantly different from diabetic control, *p <* 0.001 ^$$^Significantly different from TVL120, *p <* 0.01

The histopathological changes in the pancreatic islets of the different experimental groups are represented by Fig. 7. Figure 7a shows the normal pancreatic islet architecture of the non-diabetic control rat group. Degeneration and necrosis of islets are observed in the untreated diabetic group (Fig. 7b). The islets displayed a shrunken appearance due to degranulation of cells. TVL 60 mg (7C) and 120 mg (7D) as well as glibenclamide (7E) administration improved islet morphology. Quantitative analysis of islets is shown in Fig. 8. A significant (76 %) reduction in the number of islets was found in the untreated diabetic rats, compared to the non-diabetic control rats (*p <* 0.001). There were no significant differences in islet numbers between the rats treated with TVL and glibenclamide, compared to the non-diabetic group. Evaluation of islet size is shown in Fig. 9. Significant reduction in islet size was observed in the diabetic control (85 %), compared to the non-diabetic control group (*p <* 0.05). TVL and glibenclamide-treated rats did not show significantly reduced islet numbers compared to the non-diabetic control rats. However, TVL treatment significantly increased the number of pancreatic islets by about 67 %, compared to the diabetic control group (*p <* 0.05). TVL120 also showed a significant 61 % increase in islet number, compared to the glibenclamide group. Islet size (area μ m^2^) was significantly increased by 71 %, 73 % and 50 % respectively, following TVL 60 mg, TVL 120 mg and glibenclamide administration, compared to diabetic control group (*p <* 0.001).

### Effect of TVL on liver antioxidant enzymes, TBARS and plasma nitric oxide levels

Oxidative stress was assessed by determining the levels of antioxidant enzymes; superoxide dismutase (SOD), catalase, and glutathione peroxidase (GPx) as well as thiobarbituric acid reactive substance (TBARS), a measure of lipid peroxidation in the liver. Liver antioxidant enzyme (SOD, GPx and catalase), liver TBARS and plasma nitric oxide levels in the various groups are shown in Table 3. The untreated diabetic rats showed significantly reduced liver SOD, GPx (*p <* 0.01) and catalase (*p <* 0.05) activities compared to the non-diabetic control group. Treatment with 60 mg dose of TVL significantly enhanced the activity of SOD (*p <* 0.05), catalase (*p <* 0.05) and GPx (*p <* 0.05). The 120 mg dose of TVL produced significant increases in SOD (*p <* 0.05), catalase (*p <* 0.05) and GPx (*p <* 0.01) activities. Glibenclamide treatment produced significant increases in SOD (*p <* 0.01); GPx (*p <* 0.01) and catalase activities (*p <* 0.05), compared to the diabetic control group. There were no significant differences in antioxidant activity between the treated diabetic groups compared to the non-diabetic control group.

**Table 3:** Effect of TVL on liver antioxidant enzymes, TBARS and plasma nitric oxide levels in rats

Parameters	Experimental groups				
	NDC	DC	D + TVL 60	D + TVL 120	D + glibenclamide
Liver parameters:					
SOD (% Inhibition)	89.88 ± 2.71	43.82 ± 4.81**	72.71 ± 5.89^#^	74.08 ± 3.26^#^	80.56 ± 7.35^##^
GPx (mU/ml)	6.62 ± 0.23	1.46 ± 0.57**	4.03 ± 0.75^#^	6.44 ± 0.47^##^	6.28 ± 0.86^##^
Catalase (mU/ml)	0.34 ± 0.08	0.13 ± 0.02*	0.44 ± 0.05^#^	0.42 ± 0.08^#^	0.61 ± 0.07^#^
TBARS (nmol/mg tissue)	4.80 ± 1.65	9.86 ± 0.0.59*	7.18 ± 0.39^#^	5.53 ± 1.27^#^	7.84 ± 0.97
Plasma NO (uM)	32.61 ± 1.18	24.35 ± 0.97**	30.44 ± 1.64^#^	34.85 ± 3.31^#^	30.74 ± 3.66^#^

Non-diabetic control (NDC) and diabetic control (DC); TVL 60 mg/kg.b.w. TVL 120 mg/kg.b.w. (D + TVL 120) and glibenclamide 10 mg/kg.b.w. (D + glibenclamide). All data are expressed as mean ± SEM for 7 animals per group

*Significantly different from non-diabetic control *p <* 0.05

**Significantly different from non-diabetic control *p <* 0.01

^#^Significantly different from diabetic control *p <* 0.05

^##^Significantly different from diabetic control *p <* 0.01

The diabetic control group showed a significant 51 % increase in liver TBARS levels, compared to the non-diabetic control group (*p <* 0.05). There were no significant differences in TBARS levels between the TVL60, TVL120 and glibenclamide treated groups when compared to the non-diabetic group. However, administration of TVL 60 and 120 mg/kg produced significant reductions in liver TBARS levels, compared to the diabetic control group (*p <* 0.05).

Plasma nitric oxide levels decreased significantly in the diabetic control group, compared to the non-diabetic control group (*p <* 0.01). The diabetic groups treated with TVL60mg/kg, TVL 120 mg/kg, and glibenclamide showed significant increases in plasma NO, compared to the diabetic control group (*p <* 0.05). There were no significant differences between the treated diabetic groups and the non-diabetic control rats.

### Effect of TVL on serum lipids and liver function enzymes

Serum lipid and liver enzyme levels in the various groups are shown in Table 4. The diabetic control group showed a significant increase in triglyceride levels, compared to the non-diabetic group (*p <* 0.05). The diabetic rats treated with TVL 60, TVL 120 and glibenclamide showed significant decreases in serum triglyceride levels, compared to the diabetic control rats (*p <* 0.05). The triglyceride levels of the insulin treated group did not differ significantly from those of the diabetic control group.

**Table 4:** Effect of TVL on serum lipids and liver enzymes in rats

Parameters	Experimental groups				
	NDC	DC	D + TVL 60	D + TVL 120	D + glibenclamide
Triglycerides (mg/dl)	92.00 ± 10.58	134.50 ± 7.09*	100.70 ± 6.94^#^	93.14 ± 10.00^#^	93.50 ± 7.99^#^
Total cholesterol (mg/dl)	73.00 ± 2.48	82.25 ± 1.80	74.80 ± 5.20^#^	73.17 ± 4.45^#^	67.67 ± 4.70^#^
HDL-cholesterol (mg/dl)	24.40 ± 1.29	19.25 ± 0.75*	26.00 ± 1.58^#^	26.80 ± 1.86^#^	20.33 ± 2.08
LDL-cholesterol (mg/dl)	30.30 ± 1.66	36.10 ± 3.17	28.00 ± 1.88	27.67 ± 2.84	28.47 ± 3.14
VLDL (mg/dl)	18.40 ± 1.64	26.90 ± 1.42*	20.13 ± 1.39^#^	18.63 ± 2.00^#^	18.70 ± 1.60^#^
ALT (U/l)	62.60 ± 4.40	120.80 ± 12.52***	78.50 ± 6.83*^##^	77.75 ± 3.97* ^##^	83.25 ± 3.82*^#^
AST (U/l)	76.25 ± 1.25	98.00 ± 2.30*	78.83 ± 2.32^#^	79.83 ± 2.46^#^	81.00 ± 2.58^#^

Non-diabetic control (NDC) and diabetic control (DC); TVL 60 mg/kg.b.w., (D + TVL 60 mg/kg.b.w.TVL 120 mg/kg.b.w.(D + TVL 120) and glibenclamide 10 mg/kg.b.w.(D + glibenclamide). All data are expressed as mean ± SEM for 7 animals per group

*Significantly different from non-diabetic control *p <* 0.05

***Significantly different from non-diabetic control *p <* 0.001

^#^Significantly different from diabetic control *p <* 0.05

^##^Significantly different from diabetic control *p <* 0.01

Total cholesterol levels in the untreated diabetic rats were significantly increased, compared to the non-diabetic group (*p <* 0.05). The diabetic rats treated with TVL 60 and 120 mg/kg as well as glibenclamide, showed significantly decreased total cholesterol levels compared to the diabetic control group (*p <* 0.05). HDL-cholesterol levels were significantly reduced in the diabetic rats, compared to the non-diabetic rats (*p <* 0.05). Treatment with TVL 60 and 120 mg/kg significantly increased HDL-cholesterol levels, compared to the diabetic control rats (*p <* 0.05). There were no significant differences between the glibenclamide treated groups, compared to the diabetic control group. The HDL-cholesterol levels of the treated diabetic groups did not differ significantly from the non-diabetic group. Although there were no significant differences (*p >* 0.05) in LDL-cholesterol levels between the different experimental animal groups, the diabetic control group showed a tendency towards increased LDL-cholesterol levels compared to the other groups.

The diabetic control group showed a significant increase in VLDL-cholesterol levels, compared to the non-diabetic group (*p <* 0.05). The diabetic rats treated with TVL 60,TVL 120 and glibenclamide showed significantly decreased VLDL-cholesterol levels, compared to the diabetic control rats(*p <* 0.05). The VLDL-cholesterol levels of the treated diabetic rats did not differ significantly from the non-diabetic control group.

Serum ALT increased significantly in the diabetic control group, compared to the non-diabetic control group. TVL 60 mg/kg, TVL 120 mg/kg (*p <* 0.01) and glibenclamide-treated (*p <* 0.05) groups showed significantly increased ALT levels, compared to the non-diabetic group; but significantly reduced ALT levels compared to the diabetic control group. AST levels were significantly increased in the untreated diabetic group, compared to the non-diabetic group whereas TVL and glibenclamide treatments significantly reduced serum AST levels compared to the diabetic control group (*p <* 0.05).

## Discussion

The present study investigated the effects of a medicinal plant, *T.violacea*, on blood glucose, serum lipids and body antioxidant status in a streptozotocin (STZ)-induced diabetic rat model. STZ is widely used in experimental animals to induce diabetes due to its toxic effect on the β-cells of pancreatic islets, resulting in a loss of insulin secretion [33]. STZ-induced diabetes is characterized by hyperglycaemia, severe body weight loss, polydipsia, polyphagia and polyuria. In the present study, we found significantly elevated blood glucose levels, reduced body weights and increased water and food consumption along with increased urine output (data not shown) in the diabetic rats compared to the non-diabetic rats, indicating that diabetes was effectively induced. Treatment of diabetic rats with 60 and 120 mg/kg of TVL significantly reduced water intake but did not produce significant reductions in polyphagia (Table 2). Nevertheless, TVL treatment significantly reduced fasting blood glucose levels (Fig. 2) And improved glucose tolerance (Fig. 3), demonstrating its hypoglycaemic effects in diabetic rats.

It is known that loss of body weight and decreased growth rate in diabetic rats, despite increased food intake is due to increased catabolism of protein as a result of insulin deficiency which produces degeneration of structural proteins and muscle wasting [1]. Treatment with TVL (120 mg/kg) improved body weight suggesting that the higher dose of TVL could be protective against the degradation of structural proteins, possibly due to improved glycemic control. The present study also showed increased liver and kidney to body weight ratios in the untreated diabetic rats. Liver hypertrophy could be attributed to hypoinsulinaemia-induced influx of fatty acids, and decreased lipoprotein secretion from the liver, leading to triglyceride accumulation [34]. On the other hand, TVL treatment significantly reduced liver/body weight ratios compared to the diabetic rats, which may be attributed to the reduced triglyceride levels observed in the TVL-fed groups. Our finding relating to the kidney is not surprising since increased kidney weights in diabetes have been attributed to increased protein synthesis and lipogenesis [1]. According to Eleazu et al. (2013) [1], renal hypertrophy is considered to be early indicator of glomerular pathology associated with diabetes.

The reduced kidney/body weight ratio observed in the TVL-treated diabetic rats could therefore, demonstrate potential renoprotective effects of TVL administration. Insulin deficiency or insulin resistance results in elevated fasting and postprandial blood glucose levels [35]. However, TVL treatment produced a significant hypoglycaemic effect by reducing fasting glucose levels (Fig. 2) and improving glucose tolerance (Fig. 3). We also found significantly increased plasma insulin levels in the TVL and glibenclamide treated groups (Fig. 5). Histopathological examination of the pancreatic islets showed extensive damage to islet cells and reduction in number and size of islets in the untreated diabetic group (Fig. 7b). TVL (Fig. 7c and d) and glibenclamide-treatment (Fig. 7e) improved the morphology, number and size of islet suggesting possible protective effects of these treatments against STZ-induced islet damage which may consequently have enhanced insulin secretion as evidenced by increased plasma levels. Insulin is known to stimulate glycogen synthase and inhibit glycogenolysis in the liver, the key site for endogenous glucose production [10, 36]. Insulin deficiency therefore, results in an inactivation of glycogen synthase and promotes glycogenolysis, thereby decreasing liver glycogen content in diabetic rats [36, 37]. Our results showed that administration of both doses of TVL and glibenclamide significantly increased plasma insulin and liver glycogen levels (Fig. 5 and 6), compared to the untreated diabetic rats, which may suggest that administration of TVL and glibenclamide stimulated insulin secretion from remnant pancreatic β-cells, thereby enhancing the impaired capacity of the liver to synthesize glycogen.

Several studies have shown that *Allium sativum* lowers blood glucose in diabetic rats, and that this hypoglycaemic effect is largely due to the sulfur-containing compounds present in the plant [38, 39]. Some of the postulated mechanisms underlying the hypoglycaemic action of garlic include, increased insulin secretion from pancreatic β-cells or its release from bound insulin, combination with compounds such as cysteine to potentiate serum insulin secretion, enhanced insulin sensitivity and/or increased liver glycogen synthesis, as well as reduced oxidative stress which ameliorates pancreatic β-cell damage [29, 38–40]. It is likely that the organosulfur compounds present in TVL have similar mechanisms of action in effecting hypoglycaemia in diabetic rats. Treatment of diabetic rats with the standard hypoglycaemic drug, glibenclamide also reduced fasting blood glucose levels, but did not improve glucose tolerance. Glibenclamide has been shown to exert its hypoglycaemic effect through stimulation of insulin secretion from remnant pancreatic islet β-cells, and inhibition of glucagon secretion [41].

In this study, we also examined the role of TVL on oxidative stress in diabetes. Oxidative stress, due to increased free radical formation and reduced antioxidant status, is widely believed to be a key factor in the pathogenesis and progression of diabetes [3, 39]. Available evidence strongly suggests that excessive free radicals, particularly reactive oxygen species (ROS) generated from hyperglycemia induced glucose autoxidation and protein glycosylation, play a critical role in diabetes [29]. STZ induces oxidative stress and reduces antioxidant defense mechanisms in blood and tissues, particularly the liver [29]. The hepatotoxic effect of STZ is believed to be mediated by ROS through induction of lipid peroxidation in the hepatocellular membrane, resulting in altered permeability and loss of membrane integrity [29]. Consistent with this, we found greatly reduced levels of key antioxidant enzymes, SOD, CAT and GPx. as well as elevated lipid peroxidation as evidenced by increased TBARS levels in the liver of the diabetic control rats (Table 3), an indication of STZ-induced oxidative stress. However, TVL treatment at both 60 and 120 mg/kg doses, produced significantly increased SOD, catalase and GPx activity accompanied by decreased TBARS levels in liver, demonstrating the ability of TVL to reduce oxidative stress in diabetic rats. TVL has been shown to reduce oxidative stress in a number of previously published studies [22–24]. Moreover, garlic has been reported to exert antioxidant effects by scavenging ROS and increasing SOD, catalase and GPx. levels in cells [29]. The antioxidant activity of garlic has been mainly attributed to the presence of organosulfur compounds, which are believed to possess powerful antioxidant properties and capable of stimulating liver antioxidant enzymes [42, 43]. Our GC-MS analysis of TVL revealed the presence of disulfides, (Table 1) which have known antioxidant effects and could therefore contribute towards the antioxidant effects of TVL.

Hyperglycaemia and hyperlipidemia, two characteristic features of diabetes are believed to be associated with inhibition of endothelial nitric oxide synthase (eNOS) and consequently decreased NO production as well as increased ROS production [44]. This may lead to endothelial dysfunction and eventually vascular damage in the diabetic state as NO release from the endothelium plays an important role in regulating vascular tone [45, 46]. Notably, we also found significantly increased plasma NO levels following TVL administration as NO-generating compounds have been reported to ameliorate hyperglycaemia and oxidative stress in diabetic rats [47].

Abnormal lipid metabolism, leading to accumulation of plasma LDL, VLDL and total cholesterol as well as decreased HDL-cholesterol, is commonly associated with DM [37]. Elevated levels of LDL, VLDL and TC are considered major risk factors for cardiovascular disease (CVD). Conversely, increased HDL-cholesterol which plays a key role in cholesterol transport from the periphery to the liver reduces the risk of CVD [48]. The liver is involved in the uptake and metabolism of free fatty acids, as well as synthesis of cholesterol, triglycerides and phospholipids [49]. Normally, triglycerides are hydrolyzed by the enzyme lipoprotein lipase which is activated by insulin. However, in the diabetic state, lipoprotein lipase is not activated due to insulin deficiency which results in increased hepatic synthesis of triglycerides and an imbalance in the release and rate of clearance of VLDL-cholesterol by lipoprotein lipase [44]. Consistent with this, we found elevated triglyceride, total cholesterol, VLDL-cholesterol and decreased HDL-cholesterol levels in the untreated diabetic rats. Treatment with TVL (60 and 120 mg/kg) produced significant reductions in serum triglyceride, total cholesterol levels and VLDL-cholesterol levels, as well as increased HDL-cholesterol levels. The reduced total cholesterol and increased HDL-cholesterol levels following TVL administration are noteworthy as it has been reported that most drugs used in the treatment of hypercholesterolaemia reduce both total and HDL-cholesterol levels [48]. Sulfonylurea treatment has been found to decrease serum triglycerides and consequently, VLDL levels due to improved glycemic control [50]. The reductions in the serum levels of these lipids following glibenclamide treatment support these findings. The exact mechanisms whereby TVL exerts its hypolipidemic effects are not known but could possibly be due to the organosulfur compounds present in TVL.

Elevated levels of liver transaminases, alanine aminotransferase (ALT) and aspartate aminotransferase (AST) are considered biomarkers of hepatocellular damage, associated with fatty liver disease and hyperglycaemia in diabetes [33, 44, 51]. TVL treatment significantly reduced ALT and AST levels, suggesting that TVL may ameliorate STZ-induced hepatocyte injury in diabetic rats.

## Conclusions

Our results show that administration of TVL methanolic extract produces hypoglycaemic, antioxidant and hypolipidemic effects in diabetic rats, thus demonstrating its potential benefits in ameliorating some of the complications associated with diabetes. Further studies are required to identify the bioactive compounds present in TVL as well as to elucidate the mechanisms whereby TVL exerts its beneficial effects.
